# "Complete" and "Incomplete" Operations

**Published:** 1898-02-19

**Authors:** 


### "COMPLETE" AND "INCOMPLETE" OPERATIONS.

In regard to the question of nomenclature we would offer a word of protest against the growing custom of speaking of operations as "complete" or "incomplete," as "adequate" or "inadequate." Medical men know what these terms mean and make no mistake, but it is different with the public, and we cannot but see that it might become a serious matter if a surgeon were sued for malpraxis, and if evidence were forthcoming that the operation which he had done was well recognised in the profession as being an "incomplete" or "inadequate" one. Nothing would be more grateful to many an advocate than to hold up to obloquy the man who, having been employed by his patient to do his best for her, having been trusted by her, and having received a big fee that he might relieve her of her disease, had ended by doing what, in the words of the medical witnesses, was described as an incomplete and inadequate operation Nor do we speak merely from the point of view of expediency. When an operator does what he intended to do, surely his operation cannot be called incomplete! When it removes what it was intended to remove, surely it cannot be spoken of as inadequate! Yet some would have it so. It must, then, be asked what more does a man mean when he speaks of his operation as "complete." However far he goes he has no knowledge that the disease has not already extended farther, and the very fact that so many patients, after these "complete" operations, have died of internal cancer, is enough to show how far from complete they have been if the word may be taken as meaning the entire removal of the disease. There are, moreover, certain ethical difficulties connected with the adoption of such terms, and still more with the far too common use of the word "cure" in regard to those cases in which the disease does not return for three years. This matter is especially serious in regard to its influence on the public. Can we wonder that people ask to be operated on by men who profess to do "complete" operations, and avoid those whose methods are dubbed "incomplete" and, according to some, "inadequate"? What is in a word? we may ask. But a great deal is in a word when matters come to such a pass that, as Sir Thomas Smith remarked, some surgeons are said to be able, and others not to be able, to cure cancer. "Cure" is a dangerous word so far as ethics are concerned, for it is the one word above all others that has a fascination for the poor sufferers from this disease.

